# Supplementary material for: “Have You Seen This Drivel?” A Comparison of How Common Health Issues Are Discussed within Brachycephalic and Non-Brachycephalic Dog Breed Groups on Social Media
Source: Animals (Basel). 2024 Feb 28;14(5):757. doi: 10.3390/ani14050757 (PMC10930683; doi:10.3390/ani14050757)
Supplement: Supplementary file 1 [file animals-14-00757-s001.zip › animals-2855608-supplementary.pdf]

# Supplementary Materials: “Have You Seen This Drivel?” A Comparison of How Common Health Issues Are Discussed within Brachycephalic and Non-Brachycephalic Dog Breed Groups on Social Media

**Kitty Phillips, Carrie Stewart, Taryn Johnston and Daniel S. Mills**

**Table S1**. Key common Facebook group rules

| Key Group Rules | Brachycephalic Frequency | Non-Brachycephalic Frequency |
|---|---|---|
| Be kind and courteous: We’re all in this together to create a welcoming environment. Let’s treat everyone with respect. Healthy debates are natural, but kindness is required. | 3 groups | 5 groups |
| No hate speech or bullying: Make sure that everyone feels safe. Bullying of any kind isn't allowed, and degrading comments about things such as race, religion, culture, sexual orientation, gender or identity will not be tolerated. | 2 groups | 4 groups |
| No promotions or spam: Give more to this group than you take. Self-promotion, spam and irrelevant links aren't allowed. | 3 groups | 2 groups |
| Respect everyone’s privacy: Being part of this group requires mutual trust. Authentic, expressive discussions make groups great, but may also be sensitive and private. What's shared in the group should stay in the group. | 2 groups | 2 groups |
| No sales, advertising, breeders or rehoming: This includes sales and advertising of puppies, stud dogs and all goods. Facebook also classes rehoming of dogs as sales of animals, therefore rehoming posts will also not be approved. | 3 groups | 6 groups |
| Training recommendations: We advocate positive reinforcement training methods. We do not condone or allow aversive methods that inflict or involve punishment such as E-Collars or Prong Collars (this list is not finite). | 1 group | 3 groups |
| Contributions towards veterinary fees/fundraising: We do not allow members to contribute towards veterinary treatment as this discourages members from taking insurance cover and opens the group to begging. It’s not allowed, period. You will be removed if you suggest it or suggest anything surrounding ‘helping’ members with veterinary costs. "Go Fund Me" advertising will NOT be approved. | 2 groups | 2 groups |

| | | |
|---|---|---|
| Veterinary advice: This group is not to be used in place of veterinary advice. If you are concerned about your pet, we advise that you contact your veterinary practice/out of hours service. | 1 group | 5 groups |
